# Favorable survival of patients with nasopharyngeal carcinoma associated with metachronous multiple primary cancers: A retrospective study

**DOI:** 10.3892/ol.2025.15296

**Published:** 2025-09-23

**Authors:** Xin Huang, Ying Kong, Tianyu Wu, Zhen Meng, Min Kang

**Affiliations:** 1 Department of Radiation Oncology, The First Affiliated Hospital of Guangxi Medical University, Nanning, Guangxi 530021 P.R. China; 2 Department of Oncology, Ya'an People's Hospital, Ya'an, Sichuan 625000, P.R. China; 3 The First Clinical Medical College, Guangxi Medical University, Nanning, Guangxi 530021 P.R. China; 4 Department of Oncology, The Second Affiliated Hospital of Shandong First Medical University, Tai'an, Shandong 271000, P.R. China; 5 Guangxi Key Laboratory of Immunology and Metabolism for Liver Diseases, Guangxi Medical University, Nanning, Guangxi 530021, P.R. China; 6 Key Laboratory of Early Prevention and Treatment for Regional High Frequency Tumor (Guangxi Medical University), Ministry of Education, Nanning, Guangxi 530021, P.R. China

**Keywords:** multiple primary cancers, nasopharyngeal carcinoma, synchronous and metachronous malignancies, clinical characteristics, prognosis

## Abstract

Patients successfully treated for nasopharyngeal carcinoma (NPC) are at an increased risk of developing multiple primary cancers (MPCs), but the prognostic implications of synchronous (sMPC) vs. metachronous (mMPC) disease remain unclear. The present study aimed to compare the clinical characteristics and survival outcomes between patients with NPC with MPC, sMPC and mMPC, and to identify prognostic factors. Data from 306 patients with NPC-related MPC admitted to the First Affiliated Hospital of Guangxi Medical University (Nanning, China) from 2012 to 2023 were retrospectively analyzed, with 35 cases of sMPC (diagnosed within 6 months of the detection of the primary cancer) and 271 cases of mMPC (diagnosed >6 months apart). The clinical characteristics, treatment details and survival outcomes of patients were compared using the χ^2^ test, Fisher's exact test, t-test, Kaplan-Meier method and multivariate Cox regression analysis. The results demonstrated that sMPC, compared with mMPC, was significantly associated with a higher proportion of other-cancer-first cases (37.1 vs. 11.8%; P<0.001), an older mean age (53.3±12.4 vs. 46.2±11.9 years; P=0.001), being a heavy smoker (11.4 vs. 3.0%; P=0.042) and advanced-stage first cancers (80.0 vs. 53.5%; P=0.002). The median survival duration for the first cancer was significantly shorter in patients with sMPC compared with those with mMPC (41.3 vs. 160.3 months; P<0.001). Multivariate analysis identified mMPC [adjusted hazard ratio (HR), 0.21; P<0.001], an older age at first diagnosis (adjusted HR, 1.04; P<0.001), advanced tumor-node-metastasis (TNM) stage (adjusted HR, 2.8; P<0.001) and radiotherapy for first cancer (adjusted HR, 0.55; P=0.042) as independent prognostic factors. NPC with mMPC was associated with longer survival than NPC with sMPC. Therefore, age, TNM stage and radiotherapy were identified as key prognostic indicators. These findings highlight the need for tailored surveillance and treatment strategies to improve outcomes in this unique patient population.

## Introduction

Due to the continuous advancement of modern medical technology, the survival of patients with cancer has been markedly prolonged. However, this progress has also led to a corresponding rise in the risk of developing other primary malignancies (1–3). Multiple primary cancers (MPC) denote the diagnosis of ≥2 primary malignant tumors in a patient, either simultaneously or sequentially (4). The etiology of MPC is complex and may be associated with numerous factors, including patient genetics, immune dysregulation, exposure to carcinogens, environmental factors, treatment modalities and increased disease surveillance (5–10). In clinical practice, the diagnosis and treatment of MPC present numerous challenges (4,11). Its symptoms and imaging features closely mimic those of metastatic cancer, often leading to misdiagnosis or delayed diagnosis, which may delay the optimal timing for treatment. Furthermore, the treatment of MPC lacks standardized guidelines, and clinical decision-making requires a comprehensive consideration of several factors, including the physical condition of the patient, type of tumor pathology, tumor staging and prior treatment history, further complicating clinical decision-making (4,5,12).

Nasopharyngeal carcinoma (NPC) is a prevalent malignancy localized to the head and neck region. It is characterized by a distinct geographical distribution, with notably higher incidence rates in Southeast Asia and Southern China compared with other regions worldwide (13,14). Despite advancements in diagnostic and therapeutic technologies, NPC remains a marked public health burden due to its high incidence and mortality rates (15). Notably, the incidence of MPC among patients with NPC is relatively high (1.9–8.7%) (16–19), which may be attributed to the unique biological behavior of NPC and its treatment modalities (radiotherapy and/or chemotherapy), both of which may potentially induce second primary cancers (17,20). When patients with NPC develop MPC, clinical management and prognosis become even more complex. Therefore, a deeper understanding of the clinical characteristics and survival outcomes of patients with NPC-related MPC (NPC-MPC) is crucial for optimizing treatment strategies and improving prognosis.

Although previous studies have explored the incidence and outcomes of MPC among specific groups of cancer survivors (5,18,21,22), certain reports have indicated that patients with metachronous MPC (mMPC) have an improved prognosis compared with patients with synchronous MPC (sMPC) (4,23–26). The number of studies specifically focused on NPC-MPC cases remains limited and it is unclear whether the previously reported survival advantage of mMPC compared with sMPC is also applicable to patients with NPC in association with other primary tumors. Additionally, there is no consensus on the clinical characteristics, prognosis and prognostic factors of this complex and unique patient population. Therefore, identifying prognostic factors in patients with NPC-MPC is of critical clinical importance. These factors will not only facilitate accurate patient assessment and personalized treatment planning but also provide evidence-based guidance for clinicians to optimize therapeutic strategies. For example, identifying high-risk patient groups may help guide early detection and intervention, with the potential for improved patient prognosis.

Given the current knowledge gaps and clinical needs in the field of NPC-MPC, the present retrospective study aimed to analyze the clinical data of 306 patients with NPC-MPC, with a focus on comparing the clinical characteristics, treatment responses and survival outcomes between patients with sMPC and mMPC. Survival and multivariate regression analysis were employed to identify key prognostic factors, with the aim to provide a theoretical foundation for the clinical management of NPC-MPC cases, optimize treatment strategies and ultimately improve patient prognosis and quality of life.

## Materials and methods

### Clinical subjects

The clinical data of patients with NPC [International Classification of Diseases, Tenth Revision code C11 (27)] who were admitted to the First Affiliated Hospital of Guangxi Medical University (Nanning, China) from January 2012 to December 2023 were retrospectively collected by accessing patient medical records whilst following the appropriate research criteria and guidelines. The clinical data of patients who had been initially diagnosed with NPC-MPC were then extracted.

The diagnosis of MPC was based on the criteria established by Warren and Gates in 1932 (28). The inclusion criteria were as follows (29): i) Each tumor must be pathologically confirmed as malignant; ii) each tumor must possess distinct pathological characteristics; iii) tumors should be located in different organs or, if in the same organ, be non-contiguous; and iv) one of the tumors must be pathologically diagnosed as NPC. Furthermore, the exclusion criteria were as follows: i) No pathological evidence; ii) recurrence or metastasis of the primary cancer; and iii) incomplete clinical data. Based on the time interval between the onset of the two primary tumors, patients were further categorized into two groups: i) sMPC group, defined as those occurring simultaneously or within 6 months of each other; and ii) mMPC group, defined as those with a diagnostic interval of >6 months (30). Based on the cancer onset sequence, the NPC first (NCF) and the other cancer first (OCF) groups were defined. In NCF MPCs, NPC was the first-occurring tumor, and in OCF MPCs, other malignancies occurred before NPC. For sMPCs, classification relied on the earliest documented evidence (pathological confirmation, radiological suspicion or symptom onset). For cases with identical diagnostic timestamps (truly simultaneous presentations), precedence was assigned to the malignancy demonstrating a more advanced tumor-node-metastasis (TNM) stage. Persisting diagnostic uncertainties were resolved by symptom chronology analysis or multidisciplinary tumor board consensus.

The present retrospective study was reviewed and approved by the Medical Ethics Committee of the First Affiliated Hospital of Guangxi Medical University. As it was a retrospective study, the Ethics Committee waived the requirement for informed patient consent.

### Clinical data collection

The inclusion and exclusion criteria were applied to the patient cohort and 35 sMPC and 271 mMPC cases were selected for retrospective analysis. The clinical characteristics of patients assessed included age, sex, marital status, smoking and alcohol consumption status, family history of cancer, histological subtypes of cancer, TNM stage, treatment details and survival time. Marital status was classified as either married or unmarried. Based on the World Health Organization (WHO) classification, the histological subtypes of NPC included keratinizing squamous cell carcinoma (WHO I subtype), differentiated non-keratinizing squamous cell carcinoma (WHO II subtype) and undifferentiated non-keratinizing squamous cell carcinoma (WHO III subtype) (31). Smoking status was classified as never smoker, current or ex-smoker or heavy smoker (≥20 pack-years) (32). Alcohol consumption status was categorized into never drinker, current or ex-drinker and heavy drinker (≥30 g/day) (33). A total of two experienced pathologists independently reviewed all pathology slides to confirm the diagnosis. Discrepancies were resolved by achieving consensus. To ensure quality control, diagnostic reports were cross validated with institutional cancer registry data.

### Treatment

The clinical staging of NPC was determined according to the 2017 Eighth Edition of the American Joint Committee on Cancer staging system (34), where the TNM stage was defined as early-stage (stage I–II) or advanced-stage (stage III–IV). In accordance with the guidelines of the National Comprehensive Cancer Network for head and neck cancer (35), a standardized treatment plan was established according to the TNM stage of the patient.

### Endpoints and follow-up

All enrolled patients were followed up using outpatient reviews, inpatient examinations and telephone calls until the death of the patient or until March 2024. Overall survival (OS) was calculated from the time of diagnosis of the first cancer to the death of the patient or last follow-up. Patients lost to follow-up were treated as censored cases, with their survival time calculated up to the date of the last confirmed contact (such as their final hospital visit or telephone contact), meaning that their observation was ended at that point without an event being recorded. All surviving patients without adverse events were censored at the study end date (March 2024).

### Statistical analysis

The χ^2^ test was used for categorical variables when all expected cell counts were >5 or when ≤20% of cells had expected counts of ≤5; otherwise, Fisher's exact test was applied for 2×2 tables, and the Fisher-Freeman-Halton exact test (Monte Carlo method, 100,000 replicates, two-sided) was used for larger contingency tables. An unpaired (independent samples) t-test was used for continuous variables. OS was estimated using the Kaplan-Meier method, and survival outcomes between the two groups were compared via the log-rank test. To explore the factors influencing prognosis, multivariate Cox regression analysis was performed. The proportional hazards assumption was assessed using Schoenfeld residuals, and all covariates satisfied this assumption. Multicollinearity among covariates was evaluated using the variance inflation factor (VIF), where all VIF values were observed to be below the commonly accepted threshold of 5, indicating no significant collinearity. SPSS (version 27.0; IBM Corp.) and R Statistical Software (version 4.2.2; http://www.r-project.org) were used for all statistical analyses. Two-sided P<0.05 was considered to indicate a statistically significant difference.

## Results

### Clinical characteristics of patients with sMPC and mMPC

A total of 306 patients diagnosed with MPC-involving NPC were included in the present study (Table I). Among these, 35 patients (11.4%) had sMPC, whilst 271 patients (88.6%) had mMPC. The proportion of patients with other cancer as the first primary cancer (OCF group) was significantly higher in the sMPC group (37.1%) compared with in the mMPC group (11.8%), with the difference between groups being statistically significant (P<0.001). Conversely, the proportion of NPC as the first primary cancer (NCF) was notably higher in the mMPC group (88.2%) than in the sMPC group (62.9%). The mean age at diagnosis of MPC was significantly higher in the sMPC group (53.3±12.4 years) compared with in the mMPC group (46.2±11.9 years; P=0.001). No significant differences were observed between groups in terms of sex distribution (P=0.194), family history of cancer (P=0.100) or marital status (P=0.704). However, smoking history differed significantly, with a higher proportion of heavy smokers in the sMPC group (11.4%) compared with in the mMPC group (3.9%; P=0.042).

Patients in the sMPC group had a significantly higher proportion of advanced-stage first cancers (III/IV) compared with in the mMPC group (80.0 vs. 53.5%; P=0.002). No significant differences were observed in the TNM stage of the second primary cancer (P=0.587). Most patients had histological type III cancer (88.2%), with no significant difference between the sMPC and mMPC groups (P=0.492) (Table I).

### Age at occurrence of MPCs in patients with NPC

Patients with sMPC exhibited distinct age patterns at the time of cancer occurrence. The age at first cancer diagnosis was significantly higher in the sMPC group (53.3±12.3 years) compared with in the mMPC group (45.5±11.5 years; P<0.001). Conversely, the age at second cancer diagnosis did not differ significantly between the groups (53.3±12.4 vs. 54.5±10.6 years; P=0.814) (Table II).

When stratified by NCF and OCF groups, the age at first cancer diagnosis did not significantly differ within the NCF group (52.9±15.3 years for sMPC group vs. 48.6±10.4 years for mMPC group; P>0.05). However, in the OCF group, patients in the mMPC group were significantly younger at first cancer diagnosis (45.1±11.6 years) compared with those in the sMPC group (53.5±10.6 years; P=0.001). No significant differences were observed in the age at second cancer diagnosis within either the NCF or OCF groups (Table II).

### Distribution of affected sites in MPCs

The affected site distribution was based on the anatomical locations involved in the first and second primary cancers. Although both sites were reviewed for each patient, the nasopharynx was excluded from this summary as all patients had NPC as the index cancer. Statistical analysis revealed no significant differences between patients with sMPC and mMPC (P=0.940; Table III) with reference to all major affected sites of cancer (excluding the nasopharynx). The head and neck region was the most frequently involved site (29.0%), followed by the digestive (27.4%) and respiratory (24.4%) systems, as detailed in Table III.

### Visualization of survival time

**Figure 1.**
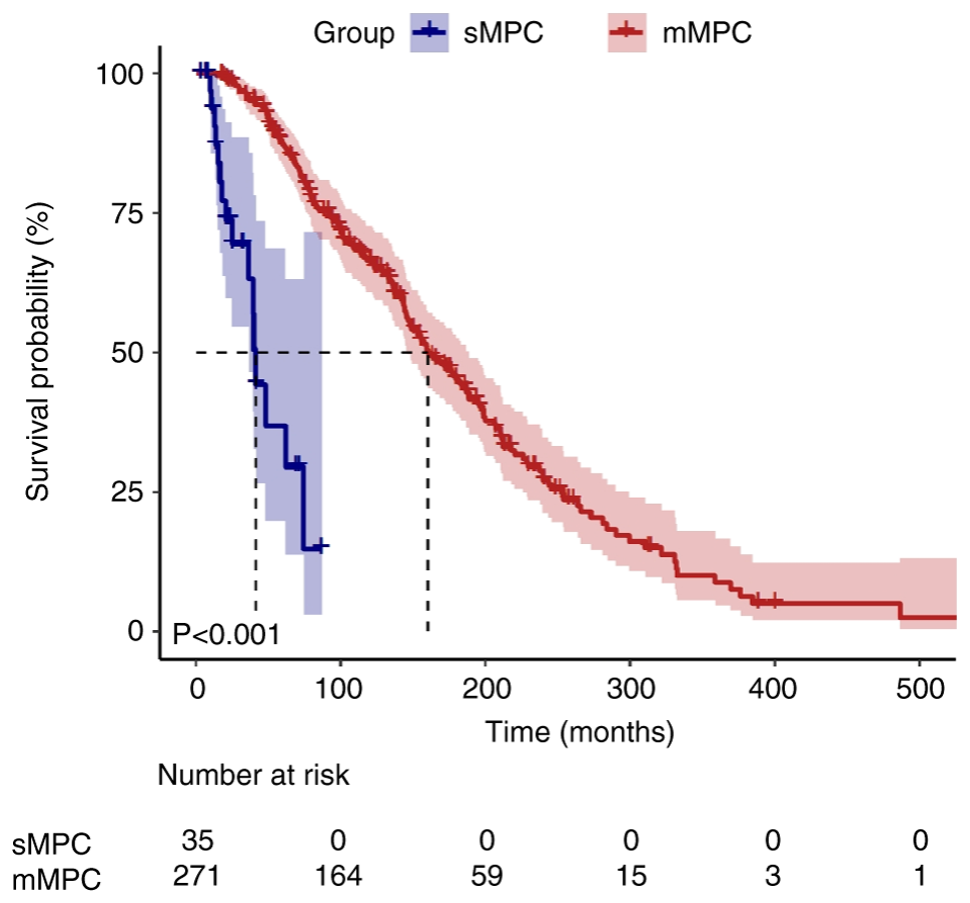
Median survival time of first cancer in patients with MPCs. Patients with mMPC demonstrated significantly improved OS compared with patients with sMPC, with median overall survival rates of 160.3 (95% CI, 139.0–181.6) and 41.3 (95% CI, 37.7–44.9) months, respectively (P<0.001), suggesting that patients with mMPC may have an improved prognosis compared with patients with sMPC. MPC, multiple primary cancers; sMPC, synchronous MPC; mMPC, metachronous MPC; CI, confidence interval.

**Figure 2.**
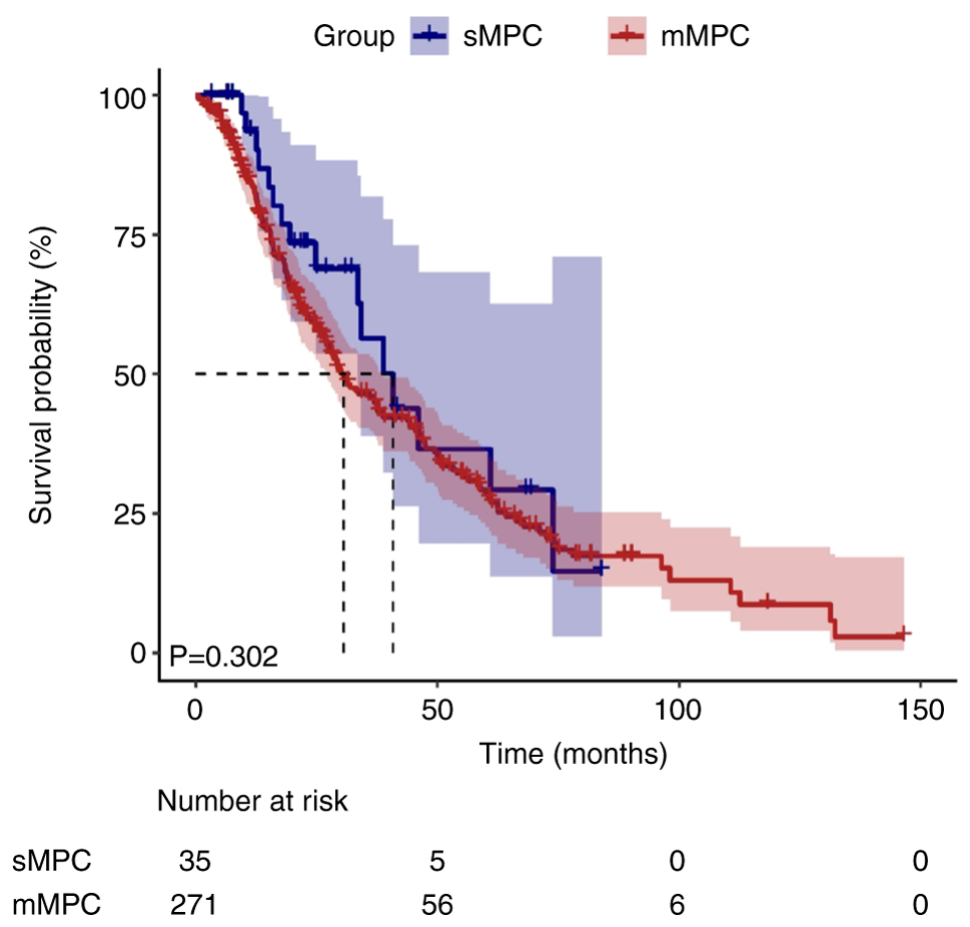
Median survival time of patients with second cancer with MPCs. The median survival time of second cancer did not differ significantly between the two groups (40.8 vs. 30.5 months; P=0.302). MPC, multiple primary cancers; sMPC, synchronous MPC; mMPC, metachronous MPC.

During the median follow-up duration of 155.2 months [95% confidence interval (CI), 137.9–172.5], 10 patients (3.3%) were lost to follow-up and censored as per the pre-defined protocol. Kaplan-Meier survival curves illustrated divergent survival outcomes between the sMPC and mMPC groups (Figs. 1 and 2). The median survival time for the first cancer was significantly shorter in the sMPC group (41.3 months; 95% CI, 37.7–44.9) compared with in the mMPC group (160.3 months; 95% CI, 139.0–181.6; log-rank P<0.001). By contrast, the median survival time for the second cancer did not differ significantly between the two groups (40.8 vs. 30.5 months; P=0.302).

### Survival outcomes and prognostic factors

#### Univariate analysis

Univariate analysis (Table IV) identified several factors that were significantly associated with first cancer survival time. Notably, an older age at first cancer diagnosis was strongly associated with shorter survival, with each 1-year increase in age corresponding to a 5% increase in the risk of death [hazard ratio (HR), 1.05; 95% CI, 1.03–1.06; P<0.001]. Smoking history also had a profound effect on survival, with heavy smokers exhibiting significantly worse survival (HR, 2.71; 95% CI, 1.37–5.38; P=0.004) compared with current or ex-smokers. Radiotherapy for the first cancer was significantly associated with improved survival (HR, 0.53; 95% CI, 0.34–0.83; P=0.011), suggesting its potential benefit for these patients. Conversely, chemotherapy was significantly associated with reduced survival, with patients receiving chemotherapy showing significantly worse survival compared with those who did not receive chemotherapy (HR, 2.01; 95% CI, 1.47–2.75; P<0.001). However, variables such as family history of cancer and marital status did not show statistically significant associations with survival (P>0.05).

### Multivariate analysis

After adjusting for confounding factors, multivariate survival analysis (Table V) revealed that mMPC was significantly associated with improved survival compared with sMPC (adjusted HR, 0.21; 95% CI, 0.11–0.40; P<0.001). Moreover, age at first cancer diagnosis was identified as an independent predictor of survival (adjusted HR, 1.04; 95% CI, 1.02–1.05; P<0.001). By contrast, advanced TNM stage (stage III/IV vs. I/II) was a significant independent predictor of poor survival (adjusted HR, 2.8; 95% CI, 1.84–4.26; P<0.001). Notably, radiotherapy for the first cancer demonstrated a protective effect (adjusted HR, 0.55; 95% CI, 0.31–0.98; P=0.042), suggesting that patients who received radiotherapy may have had improved survival outcomes. Furthermore, chemotherapy for the first primary cancer revealed a significant univariate association with a worse survival (HR, 2.01; 95% CI, 1.47–2.75; P<0.001; Table IV); however, this significance was attenuated in multivariate models (adjusted HR, 1.12; P=0.549; Table V). A similar pattern was observed for second primary cancer chemotherapy, where univariate analysis demonstrated a significant association (HR, 1.55; 95% CI, 1.15–2.10; P=0.005; Table IV) that was diminished in multivariate analysis (adjusted HR, 1.24; P=0.200; Table V).

## Discussion

The current retrospective cohort analysis delineated distinct clinicopathological patterns and survival trajectories between the sMPC and mMPC groups of MPCs in patients with NPC. The following principal findings emerged: i) Patients with sMPC exhibited unique demographic profiles characterized by older age, heavier smoking burden and advanced-stage first cancers; and ii) synchronicity independently predicted survival outcomes, with the sMPC group demonstrating a 4.8-fold increased mortality risk compared with the mMPC group. The significant differences in clinical characteristics observed between the two groups have important implications for clinical management.

The demographic divergence observed between groups suggest distinct carcinogenic mechanisms. The older age of the sMPC cohort at initial diagnosis and its elevated heavy smoking prevalence aligned with cumulative mutagenic exposure models. Notably, 37.1% of patients with sMPC developed OCF malignancies as first primaries compared with 11.8% in the mMPC group. This is similar to the results of previous studies (36,37) and implies potentially shared etiological factors such as aging, genetic mutations and tobacco-related field cancerization that simultaneously increase the risk of multiple cancers or exposure to a unique set of carcinogens that trigger the development of multiple primary tumors at the same time (7,38–40). The older age at diagnosis in the sMPC group may be due to the cumulative exposure to carcinogens over a longer period. This is consistent with the multistep carcinogenesis process theory, where genetic mutations and cellular damage accumulates over time, often due to prolonged exposure to carcinogens, gradually resulting in an association with cancer development (5,36,41,42).

The head and neck region, and the digestive and respiratory systems, were the most frequently affected sites in the secondary malignancies, which is generally consistent with previous research findings. The presence of multiple synchronous tumors in the head and neck area and the upper aerodigestive tract has been well established (16,17,43) and may be explained by the concept of ‘field cancerization’ (44). This trend may result from the growing incidence of thyroid, lung and digestive system malignancies (45–47), and the observation highlights the need for enhanced surveillance for malignancies of the head and neck and upper aerodigestive tract to be integrated throughout the treatment and management process of NPC. Such surveillance can facilitate the early detection of malignancies and help formulate personalized treatment strategies for patients with NPC exhibiting MPC.

Moreover, the survival analysis revealed that patients with mMPC had an improved prognosis compared with those with sMPC (median overall survival, 160.3 vs. 41.3 months; P<0.001). This survival disparity may reflect divergent biological pathways. In the sMPC cohort, advanced age (53.3±12.4 vs. 46.2±11.9 years; P=0.001) and heavy smoking prevalence (11.4 vs. 3.0%; P=0.042) may indicate a cumulative mutagenic exposure potentially driving synchronous carcinogenesis through field cancerization (44). This mechanism, consistent with previous findings, is associated with synchronous tumors in related regions such as the head and neck, lung and colorectal areas, often exhibiting more aggressive behavior (23–25). Additionally, patients with sMPC have a significantly higher proportion of advanced-stage first cancers (80.0 vs. 53.5%; P=0.002), aligning with findings that synchronous colorectal cancer tends to present with advanced TNM stages and larger tumor diameters. This higher intrinsic tumor burden inherently limits therapeutic options and effectiveness (25). Conversely, patients with mMPC benefit from longer inter-cancer intervals (>6 months): Tissue repair reduces prior treatment toxicity (for example, chemotherapy-related risk drops from a univariate HR value of 2.01 to an adjusted HR value of 1.12), which is consistent with the finding that metachronous colorectal cancer has fewer complications and improved tolerance due to sufficient inter-cancer intervals (25). Over time, restored immune competence and DNA repair capacity in patients with mMPC may further lower the risk of aggressive tumor progression. Crucially, regular monitoring enabled by the inter-cancer interval facilitates early detection of second primary cancers, in line with the conclusion that metachronous cancers are more likely to be diagnosed at earlier, curable stages (4). Moreover, a reduced mutual influence between tumors in mMPC facilitates radical treatments such as radiotherapy, as observed in the 85% 5-year survival rate with radiotherapy for metachronous head and neck cancers (23), mirrored by the significant benefit of radiotherapy observed in the present study (adjusted HR, 0.55; P=0.042). In summary, sMPC has a poor prognosis that is associated with field cancerization-driven synchronous progression, a high tumor burden and treatment limitations. By contrast, mMPC exhibits survival advantages from inter-cancer interval-driven bodily repair, early detection and access to radical treatments.

Results of the present multivariate analysis demonstrated that mMPC is an independent survival predictor, whilst radiotherapy was revealed to be associated with protective effects despite the complexity of MPC. This underscores its indispensable role in NPC management. The identification of independent prognostic factors, such as mMPC, age at first cancer diagnosis, TNM stage and radiotherapy for the first cancer provides valuable guidance for clinicians to stratify patients and formulate personalized treatment plans. This finding is consistent with previous studies (16,48). The protective effect of radiotherapy on patient survival is consistent with its established role in NPC treatment. Conversely, chemotherapy was observed to be significantly associated with worse survival in the univariate analysis; however, this finding lost statistical significance in the multivariate model. This discrepancy can be attributed to confounding by indication. Specifically, patients receiving chemotherapy were more likely to present with advanced-stage disease, which is itself a strong predictor of poor prognosis. Once the model was adjusted for TNM stage and other covariates, the independent effect of chemotherapy diminished, suggesting that chemotherapy use was a marker of disease severity rather than a direct cause of worse outcomes.

Clinically, the aforementioned findings advocate for tailored surveillance strategies. Universal TNM staging remains critical for all patients given the persistent prognostic impact of advanced-stage disease. For patients with sMPC who are characterized by older age, a higher prevalence of advanced-stage first cancers and heavy smoking, comprehensive baseline assessments (such as whole-body PET-CT and endoscopy) are essential to avoid missing synchronous second primary malignancies. Post-treatment surveillance should be intensified for early detection of subsequent primaries, alongside mandatory smoking cessation interventions. For patients with mMPC, long-term surveillance targeting the head and neck region and upper aerodigestive tract should commence after NPC diagnosis. Treatment strategies require a tiered optimization approach: Radiotherapy should be prioritized for the first cancer given its independent association with patient survival, whilst chemotherapy decisions warrant a cautious risk assessment that accounts for age and TNM stage disparities in sMPC whilst weighing cumulative treatment burdens in mMPC.

Although the present study provides valuable insights into the survival outcomes of patients with NPC who have MPC, there are several limitations to consider. The retrospective nature of the study may have led to selection bias, potentially affecting the accuracy and reliability of the data. Therefore, further prospective studies are needed to confirm these findings. Additionally, the focus of the study on a single cohort of patients with NPC may limit the generalizability of the results to other cancer types or populations. Future studies should explore the molecular mechanisms underlying synchronous and metachronous cancer development to inform treatment strategies.

In conclusion, the present study highlights the significant clinical heterogeneity and survival disparities between patients with NPC who also present with either sMPC or mMPC. Among patients with mMPC, a younger age, early TNM stage and radiotherapy for the first cancer are associated with improved survival outcomes. By identifying key prognostic factors and their implications for treatment, these findings can inform clinical practice and guide future research aimed at improving the outcomes for this complex patient population.

## Figures and Tables

**Table I.** Clinical characteristics of 306 patients with multiple primary cancers.

Variable	Total (n=306)	sMPC group (n=35)	mMPC group (n=271)	P-value	Test statistic
Order of occurrence				<0.001^a^	-
OCF	45 (14.7)	13 (37.1)	32 (11.8)		
NCF	261 (85.3)	22 (62.9)	239 (88.2)		
Sex				0.194	1.686
Male	216 (70.6)	28 (80.0)	188 (69.4)		
Female	90 (29.4)	7 (20.0)	83 (30.6)		
Age at MPC diagnosis, years	47.0±12.2	53.3±12.4	46.2±11.9	0.001	10.822
Family history of cancer				0.100^a^	-
No	267 (87.3)	27 (77.1)	240 (88.6)		
Yes	39 (12.7)	8 (22.9)	31 (11.4)		
Marital status				0.704^a^	-
Married	291 (95.1)	34 (97.1)	257 (94.8)		
Unmarried	15 (4.9)	1 (2.9)	14 (5.2)		
Smoking status				0.042^a^	-
Never smoker	204 (66.7)	19 (54.3)	185 (68.3)		
Current or ex-smoker	90 (29.4)	12 (34.3)	78 (28.8)		
Heavy smoker^b^	12 (3.9)	4 (11.4)	8 (3.0)		
Alcohol status				0.061^a^	-
Never drinker	231 (75.5)	21 (60.0)	210 (77.5)		
Current or ex-drinker	49 (16.0)	9 (25.7)	40 (14.8)		
Heavy drinker^c^	26 (8.5)	5 (14.3)	21 (7.7)		
TNM stage - first cancer				0.002^a^	-
I/II	129 (42.2)	6 (17.1)	123 (45.4)		
III/IV	173 (56.5)	28 (80.0)	145 (53.5)		
Unknown	4 (1.3)	1 (2.9)	3 (1.1)		
TNM stage - second cancer				0.587^a^	-
I/II	120 (39.2)	14 (40.0)	106 (39.1)		
III/IV	181 (59.2)	20 (57.1)	161 (59.4)		
Unknown	5 (1.6)	1 (2.9)	4 (1.5)		
Histological type				0.492^a^	-
I	7 (2.3)	0 (0.0)	7 (2.6)		
II	29 (9.5)	5 (14.3)	24 (8.9)		
III	270 (88.2)	30 (85.7)	240 (88.6)		
Surgery - first cancer				0.003^a^	-
No	270 (88.2)	25 (71.4)	245 (90.4)		
Yes	36 (11.8)	10 (28.6)	26 (9.6)		
Radiotherapy - first cancer				<0.001^a^	-
No	39 (12.7)	14 (40.0)	25 (9.2)		
Yes	267 (87.3)	21 (60.0)	246 (90.8)		
Chemotherapy - first cancer				0.819	0.052
No	117 (38.2)	14 (40.0)	103 (38.0)		
Yes	189 (61.8)	21 (60.0)	168 (62.0)		
Surgery - second cancer				<0.001	11.308
No	129 (42.2)	24 (68.6)	105 (38.7)		
Yes	177 (57.8)	11 (31.4)	166 (61.3)		
Radiotherapy - second cancer				0.002	9.993
No	232 (75.8)	19 (54.3)	213 (78.6)		
Yes	74 (24.2)	16 (45.7)	58 (21.4)		
Chemotherapy - second cancer				0.116	2.473
No	186 (60.8)	17 (48.6)	169 (62.4)		
Yes	120 (39.2)	18 (51.4)	102 (37.6)		
Survival state				0.047	3.942
Survived	119 (38.9)	19 (54.3)	100 (36.9)		
Dead	187 (61.1)	16 (45.7)	171 (63.1)		
Median survival time of first cancer, months	155.2 (137–172.5)	41.3 (37.7–44.9)	160.3 (139.0–181.6)	<0.001	77.794
Median survival time of second cancer, months	32.0 (26.3–37.7)	40.8 (29.0–52.7)	30.5 (24.2–36.8)	0.302	1.065

Data are presented as n (%), mean ± standard deviation or median (95% CI).

a Fisher's exact test;

b heavy smoker, ≥20 pack-years (30);

c heavy drinker, ≥30 g/day (31). OCF, other cancer first; NCF, nasopharyngeal cancer first; MPC, multiple primary cancers; sMPC, synchronous MPC; mMPC, metachronous MPC; TNM, tumor-node-metastasis; CI, confidence interval.

**Table II.** Age at occurrence of multiple primary cancers involving nasopharyngeal carcinoma.

			NCF group (n=261)		OCF group (n=45)	
				
Variable	sMPC group (n=35)	mMPC group (n=271)	sMPC	mMPC	sMPC	mMPC
Age at first cancer diagnosis, years	53.3±12.3	45.5±11.5^a^	52.9±15.3	48.6±10.4	53.5±10.6	45.1±11.6^b^
Age at second cancer diagnosis, years	53.3±12.4	54.5±10.6	53.0±15.4	54.3±11.3	53.5±10.6	54.5±10.5

a P<0.001 vs. sMPC group;

b P=0.001 vs. sMPC group. MPC, multiple primary cancers; sMPC, synchronous MPC; mMPC, metachronous MPC; OCF, other cancer first; NCF, nasopharyngeal cancer first.

**Table III.** Distribution of affected sites in synchronous and metachronous multiple primary cancers.

Affected site	Total (n=303)	sMPC group (n=36)	mMPC group (n=267)	P-value
Respiratory system	74 (24.4)	10 (27.8)	64 (24.0)	0.940
Digestive system	83 (27.4)	10 (27.8)	73 (27.3)	
Urinary system	22 (7.3)	3 (8.3)	19 (7.1)	
Reproductive system	21 (6.9)	1 (2.8)	20 (7.5)	
Head and neck	88 (29.0)	10 (27.8)	78 (29.2)	
Others	15 (5.0)	2 (5.6)	13 (4.9)	

Data are presented as n (%). This table summarizes non-nasopharyngeal anatomical sites of first and/or second primary cancers in all patients. Each patient may contribute to one or more non-nasopharyngeal anatomical site. The nasopharynx is excluded as all patients had nasopharyngeal carcinoma as the baseline diagnosis. The sMPC count exceeds its patient number by 1 as 1 patient with sMPC had two distinct non-nasopharyngeal primary sites; the mMPC count is smaller than its patient number as 4 patients with mMPC had unclassifiable or incomplete site information. The P-value was obtained using the Fisher-Freeman-Halton exact test (Monte Carlo, two sided, 100,000 replicates). MPC, multiple primary cancers; sMPC, synchronous MPC; mMPC, metachronous MPC.

**Table IV.** Univariate analysis of prognostic factors in multiple primary cancers.

Characteristic	HR (95% CI)	P-value
Order of occurrence (OCF vs. NCF)	0.64 (0.42–1.01)	0.054
Sex (Male vs. female)	0.72 (0.52–0.99)	0.036
Age at first cancer diagnosis (continuous variable)	1.05 (1.03–1.06)	<0.001
Family history of cancer (Yes vs. no)	1.05 (0.67–1.66)	0.832
Age at MPC diagnosis (continuous variable)	1.04 (1.02–1.05)	<0.001
Age at second cancer diagnosis (continuous variable)	0.99 (0.97–1.00)	0.050
Marital status (Unmarried vs. married)	0.73 (0.30–1.78)	0.470
Smoking (reference, Never)		0.005
Current or ex-smoker	1.51 (1.08–2.10)	
Heavy^a^	2.71 (1.37–5.38)	
Alcohol (reference, Never)		0.054
Current or ex-drinker	1.24 (0.82–1.89)	
Heavy^b^	1.88 (1.14–3.09)	
First cancer affected site (reference, Nasopharynx)		0.292
Respiratory system	2.77 (0.68–11.26)	
Digestive system	1.98 (1.07–3.66)	
Urinary system	1.22 (0.39–3.83)	
Reproductive system	1.27 (0.52–3.1)	
Others	1.61 (0.59–4.36)	
Secondary cancer site (reference, Nasopharynx)		0.455
Respiratory system	1.01 (0.63–1.63)	
Digestive system	1.01 (0.63–1.60)	
Urinary system	0.82 (0.36–1.86)	
Reproductive system	0.58 (0.27–1.27)	
Head and neck system	0.35 (0.10–1.14)	
Others	0.76 (0.49–1.19)	
TNM stage - first cancer (reference, I/II)		<0.001
III/IV	4.54 (3.21–6.42)	
Unknown	3.58 (1.11–11.56)	
TNM stage - second cancer (reference, I/II)		0.009
III/IV	1.62 (1.18–2.22)	
Unknown	1.28 (0.46–3.56)	
Histology type (reference, I)		0.494
II	0.72 (0.27–1.92)	
III	0.61 (0.25–1.49)	
Surgery - first cancer (Yes vs. no)	1.42 (0.88–2.29)	0.174
Radiotherapy - first cancer (Yes vs. no)	0.53 (0.34–0.83)	0.011
Chemotherapy - first cancer (Yes vs. no)	2.01 (1.47–2.75)	<0.001
Surgery - second cancer (Yes vs. no)	0.47 (0.35–0.64)	<0.001
Radiotherapy - second cancer (Yes vs. no)	1.31 (0.93–1.84)	0.131
Chemotherapy - second cancer (Yes vs. no)	1.55 (1.15–2.10)	0.005

a Heavy smoker, ≥20 pack-years (30);

b heavy drinker, ≥30 g/day (31). OCF, other cancer first; NCF, nasopharyngeal cancer first; MPC, multiple primary cancers; TNM, tumor-node-metastasis; HR, hazard ratio; CI, confidence interval.

**Table V.** Multivariate analysis of prognostic factors in multiple primary cancers.

Variable	Crude HR (95% CI)	Crude P-value	Adjusted HR (95% CI)	Adjusted P-value
MPC type (mMPC vs. sMPC)	0.11 (0.06–0.20)	<0.001	0.21 (0.11–0.40)	<0.001
Sex (Male vs. female)	0.72 (0.52–0.99)	0.036	0.89 (0.62–1.3)	0.553
Age at first cancer diagnosis (continuous variable, per year increase)	1.05 (1.03–1.06)	<0.001	1.04 (1.02–1.05)	<0.001
Smoking status (Current or ex-smoker vs. never smoked)	1.51 (1.08–2.10)	0.015	0.98 (0.68–1.43)	0.927
Smoking status (Heavy smoker^a^ vs. never smoked)	2.71 (1.37–5.38)	0.004	1.32 (0.64–2.71)	0.458
TNM stage of first cancer (III/IV vs. I/II)	4.54 (3.20–6.42)	<0.001	2.8 (1.84–4.26)	<0.001
TNM stage of first cancer (unknown vs. I/II)	3.58 (1.11–11.57)	0.033	0.58 (0.16–2.12)	0.408
TNM stage of second cancer (III/IV vs. I/II)	1.62 (1.18–2.22)	0.003	1.81 (1.24–2.63)	0.002
TNM stage of second cancer (unknown vs. I/II)	1.28 (0.46–3.56)	0.631	1.47 (0.50–4.28)	0.480
Radiotherapy for first cancer (Yes vs. no)	0.53 (0.34–0.83)	0.011	0.55 (0.31–0.98)	0.042
Chemotherapy for first cancer (Yes vs. no)	2.01 (1.47–2.75)	<0.001	1.12 (0.77–1.63)	0.549
Surgery for second cancer (Yes vs. no)	0.47 (0.35–0.64)	<0.001	0.82 (0.57–1.20)	0.311
Chemotherapy for second cancer (Yes vs. no)	1.55 (1.15–2.10)	0.005	1.24 (0.89–1.72)	0.200

a Heavy smoker (≥20 pack-years) (30). sMPC, synchronous multiple primary cancers; mMPC, metachronous multiple primary cancers; TNM, tumor-node-metastasis; HR, hazard ratio; CI, confidence interval.

## Data Availability

The data generated in the present study may be requested from the corresponding author.
